# Design, Development, and Evaluation of a Telemedicine Platform for Patients With Sleep Apnea (Ognomy): Design Science Research Approach

**DOI:** 10.2196/26059

**Published:** 2021-07-19

**Authors:** Pavankumar Mulgund, Raj Sharman, Daniel Rifkin, Sam Marrazzo

**Affiliations:** 1 State University of New York at Buffalo Buffalo, NY United States; 2 Sleep Centers of Western New York Buffalo, NY United States; 3 Buffalo Niagara Medical Campus, Inc Buffalo, NY United States

**Keywords:** design science research, telemedicine platform, sleep apnea care, mHealth, telemedicine, sleep apnea, mobile health, web application, mobile phone

## Abstract

**Background:**

With an aging population and the escalating cost of care, telemedicine has become a societal imperative. Telemedicine alternatives are especially relevant to patients seeking care for sleep apnea, with its prevalence approaching one billion cases worldwide. Increasing awareness has led to a surge in demand for sleep apnea care; however, there is a shortage of the resources and expertise necessary to cater to the rising demand.

**Objective:**

The aim of this study is to design, develop, and evaluate a telemedicine platform, called Ognomy, for the consultation, diagnosis, and treatment of patients with sleep apnea.

**Methods:**

Using the design science research methodology, we developed a telemedicine platform for patients with sleep apnea. To explore the problem, in the analysis phase, we conducted two brainstorming workshops and structured interviews with 6 subject matter experts to gather requirements. Following that, we conducted three design and architectural review sessions to define and evaluate the system architecture. Subsequently, we conducted 14 formative usability assessments to improve the user interface of the system. In addition, 3 trained test engineers performed end-to-end system testing to comprehensively evaluate the platform.

**Results:**

Patient registration and data collection, physician appointments, video consultation, and patient progress tracking have emerged as critical functional requirements. A telemedicine platform comprising four artifacts—a mobile app for patients, a web app for providers, a dashboard for reporting, and an artificial intelligence–based chatbot for customer onboarding and support—was developed to meet these requirements. Design reviews emphasized the need for a highly cohesive but loosely coupled interaction among the platform’s components, which was achieved through a *layered modular* architecture using third-party application programming interfaces. In contrast, critical findings from formative usability assessments focused on the need for a more straightforward onboarding process for patients, better status indicators during patient registration, and reorganization of the appointment calendar. Feedback from the design reviews and usability assessments was translated into technical improvements and design enhancements that were implemented in subsequent iterations.

**Conclusions:**

Sleep apnea is an underdiagnosed and undertreated condition. However, with increasing awareness, the demand for quality sleep apnea care is likely to surge, and creative alternatives are needed. The results of this study demonstrate the successful application of a framework using a design science research paradigm to design, develop, and evaluate a telemedicine platform for patients with sleep apnea and their providers.

## Introduction

### Background

The National Sleep Foundation defines obstructive sleep apnea (OSA) as a chronic condition characterized by involuntary breathing cessation while the patient is asleep. It is a common disorder that affects more than 22 million Americans [1]. A previous study estimated that OSA affects 26% of adults aged between 30 and 70 years [2]. Gender, age, family history, obesity, smoking, and alcohol use often make patients more vulnerable to OSA. Furthermore, OSA is increasingly recognized as a risk factor for medical conditions, such as hypertension, cardiovascular diseases, and stroke [3]. Therefore, it is important to diagnose and treat OSA early. Continuous positive airway pressure (CPAP) remains the gold standard for treatment [4]. For patients who do not respond to CPAP, other treatment options may be prescribed based on the severity of the condition. Increasing awareness coupled with better prognosis from timely interventions has led to an exponential surge in the demand for evaluation and treatment services, but there has not been a commensurate increase in resources to deal with this increasing demand. The diagnosis and treatment of OSA are particularly challenging from an operational perspective because of the limited number of sleep clinics [5]. To compound this problem, there is also an acute shortage of clinical expertise for delivering these services, with the ratio of general population to certified sleep specialists being as high as 43,000:1 [6]. The situation is further exacerbated by their inequitable distribution, with most specialists serving in urban areas. Therefore, there is a need to explore other approaches for diagnosing and treating patients with sleep apnea. Telemedicine is a potentially viable alternative.

In this study, we explore this alternative by developing and evaluating a telemedicine platform for the diagnosis and treatment of OSA. Specifically, we addressed the following two research questions:

- How can we conceptualize and design a patient-first telemedicine platform to provide high-quality sleep care to patients from the comfort of their homes?
- How can we evaluate the artifact’s design quality and its potential in reducing access barriers and operational bottlenecks in the diagnosis and treatment of sleep apnea?

Following the design science research (DSR) framework [7,8], we developed a telemedicine platform called *Ognomy* (North American Sleep Management Inc), where the data collection, consultation with physicians, patient education, and monitoring occur using a mobile app. Access to nursing staff, sleep technicians, and physicians is available through video consultation. Artificial intelligence (AI)–based chatbots for self-help and helpline contacts are provided to assist care seekers in using the app effectively. Such a platform is expected to reduce costs and increase access to care while maintaining quality expectations.

### Telemedicine Background

The World Health Organization defines telemedicine as “the use of information and communication technologies in the delivery of health care services, the diagnosis of a condition, prevention of diseases and injuries, treatment, research, and evaluation of patients at a distance” [9]. Owing to its advantages, the development, deployment, and use of telemedicine have surged in the United States [10] and other developed nations [11]. Recent studies have shown that telemedicine interventions effectively improve clinical outcomes [12], decrease costs by reducing expensive inpatient service utilization [13], and provide a superior patient experience [14], particularly in the areas of chronic disease management, such as diabetes [15], chronic heart diseases [16], thyroid disorders [17], varicose veins [18], stroke [19], mental and emotional health issues [20], and sexually transmitted diseases [21]. Previous research has also discussed the benefits, drawbacks, and barriers to the adoption of telemedicine. Some common barriers include patients’ poor perception of its efficacy [22], nonacceptance by insurance companies [23], and technology hurdles [24]. However, with the growing maturity of technology platforms and increasing patient acceptance, telemedicine interventions have begun to overcome these barriers. A study by Watson et al [25] found that monitoring patients via telemedicine is as effective as traditional medical processes. Furthermore, some studies [26,27] have explored the use of telemedicine to monitor compliance with CPAP therapy. However, the design of telemedicine interventions that focus on testing and diagnosing patients with OSA has not been well addressed in previous research. Our study addresses this gap by developing a comprehensive telemedicine platform that caters to all stakeholders involved in seeking and providing OSA care.

## Methods

### Overview

**Figure 1:**
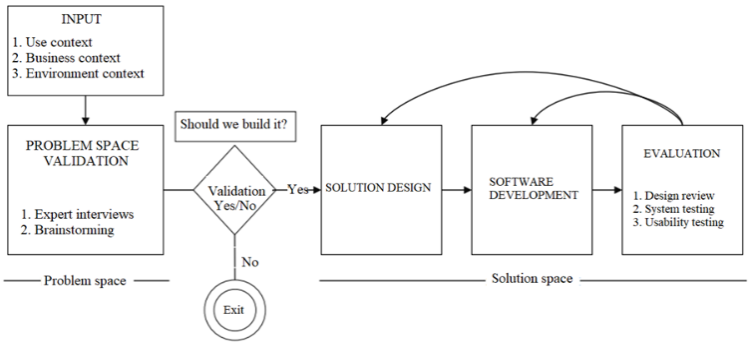
Design science research approach.

Overall, we follow a DSR approach, a principal research methodology that has evolved within the information systems (ISs) discipline. It is defined as the development of a system by an iterative process of developing and evaluating artifacts [28]. The primary goal of this study is to define and develop a telemedicine platform that caters to all stakeholders involved in diagnosing, treating, and managing sleep apnea. Previous research has discussed the process of conducting and evaluating design science [29]. We used the framework suggested by Horvath [30]. Our research approach is illustrated in Figure 1.

Furthermore, this framework divides DSR into three phases: (1) exploring the problem, the context, and the activities using activity theory; (2) designing and developing solutions; and (3) evaluating solutions and generalizing design principles.

We present the details of the abovementioned three phases in the following sections.

### Exploration of the Problem, Context, and Activities Using Activity Theory

We used activity theory to guide our understanding of the end user and business context. According to activity theory, an activity is a basic unit of analysis [31], which includes six components: (1) subjects or people involved in the activity, (2) object or intended goal of the activity, (3) tools that mediate the activity, (4) rules imposed by the business environment, (5) division of labor among various stakeholders in the organization, and (6) the community impacted by the activity. Activity theory was an apt choice for our study because it emphasizes developing a comprehensive understanding of the environment in which the system operates (which includes participants and subsystems), including all the complexity that manifests from the interactions of various actors in the real world rather than from the limited viewpoint of a single actor [32]. Furthermore, activity theory allows us to develop flexible systems that dynamically cater to various users’ specific workflows, such as patients, providers, office administrators, and information technology (IT) staff. Contemporary activity theory focuses on the interaction of several systems of activities to investigate a complex social phenomenon. In doing so, it brings to the fore some of the deeply embedded contextual issues associated with the research topic [33].

At the outset, we identified the key stakeholders (subjects) involved in the process of seeking and providing care for sleep apnea, including patients, providers, referring physicians, administrative staff such as billers and coders, and back-office personnel. To develop a deeper understanding of their roles in seeking and providing care, the resources they require, and the challenges they face, we conducted six structured interviews with each of these stakeholders and two brainstorming workshops. Details of interview participants and interview questions are presented in Multimedia Appendix 1. The dimensions of activity theory guided the questions and the line of inquiry. On the basis of interviews and brainstorming sessions, we identified Health Information Portability and Accountability Act (HIPAA) application forms, health forms, information about the diagnosis, and treatment protocols as critical resources. Enrollment, consultation, and monitoring emerged as crucial processes. In the interviews, patients expressed a strong preference for a mobile app for consultation, whereas providers favored a web portal for documentation, reporting, and integration with an electronic medical record (EMR) system. During the interviews, the collection of patient information, such as personal and contact details, HIPAA consent, insurance information, and prior health history, emerged as a crucial design challenge. The crux of the problem was to collect all relevant data from patients without overwhelming them. Owing to their limited interactivity, traditional data entry forms are not very effective in engaging users, especially when a large amount of information is collected. Guided by prior research on the effectiveness of chatbots in improving patient engagement [34,35] and care experience [36], we decided to develop a rule-based AI chatbot for enhanced engagement and better patient interaction.

In addition, stakeholders pointed out several business rules and constraints that emerged from the business context. These rules are subsequently coded into the system to develop a patient-first, state-of-the-art telemedicine platform for patients with OSA. The contextual details are presented in Table 1 along the dimensions of activity, subject, instrument, object, division of labor, community, and rules.

**Table 1:** Medical activity system mapped to technical activity system.

Activity theory construct	End user context	System construct
Subject	Patient with sleep apnea Providers Admin staff Sleep technicians Billing and patient support	Built-in customizable user interface to sign-in users Adaptive authentication
Rules	Referrals or self-referrals rules Rules of patient health status tracking Previous health or surgical history of the patient Health Information Portability and Accountability Act rules Positive air pressure compliance rules App terms and conditions	Multiple patient workflows Multiple compliance program rules Managing patient progress Maintenance of health care database records
Division of labor	Doctors: provide treatment Sleep technicians: monitor sleep laboratory Admin staff: process billing and offer patient support Insurance: review and pay claims Vendors: manufacture and ship device	Integration with reusable business services such as payment, video consultation, and role-based access control Dashboards for multiple users (physicians, patients, administrators, and technicians)
Community forum	Patients Primary care physicians Sleep specialists Administrators Management Information technology service providers CPAP^a^ vendors	Social and enterprise identity federation Integration with Facebook and Instagram Offering support services Access control for AWS^b^ resources
Instrument	Frontline Mobile app for patient Web app for doctors Virtual assistants for patient onboarding and support Dashboard to track patient progress Enabler HST^c^ device CPAP device Infrastructure AWS (cloud)	Artificial intelligence–based responsive chatbot Order management CPAP tracking HST tracking
Object	Objective: provide a telemedicine platform for the diagnosis, treatment, and management of sleep apnea Outcome: seek to improve the condition of patients with sleep apnea	N/A^d^

^a^CPAP: continuous positive airway pressure.

^b^AWS: Amazon Web Services.

^c^HST: home sleep test.

^d^N/A: not applicable.

### System Design and Development

#### Product Concept

**Figure 2:**
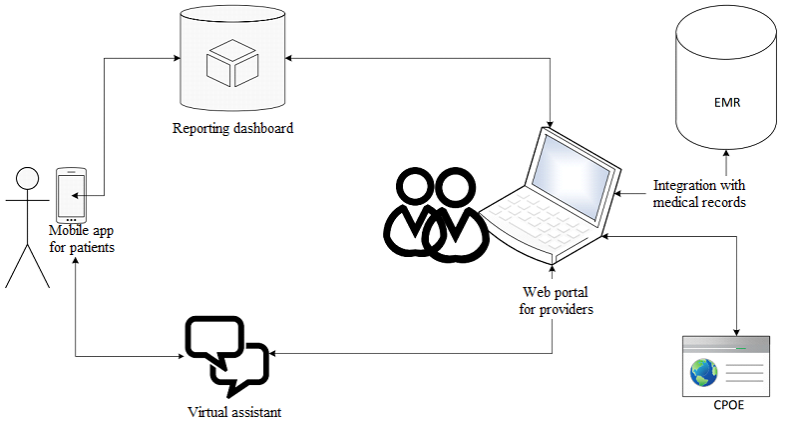
Telemedicine platform for sleep apnea. CPOE: computerized physician order entry; EMR: electronic medical records.

In this section, we describe the development of the four specific artifacts that support the needs of all stakeholders involved in seeking and providing care. These artifacts include (1) a mobile app for patients, (2) a web app for providers, (3) a dashboard for reporting, and (4) an AI-based chatbot for patient onboarding and support. This telemedicine platform is an example of a design science approach. The ecosystem of the telemedicine platform is shown in Figure 2. Our final platform, which involves four artifacts working together, offers access to high-quality care for patients with sleep apnea.

**Figure 3:**
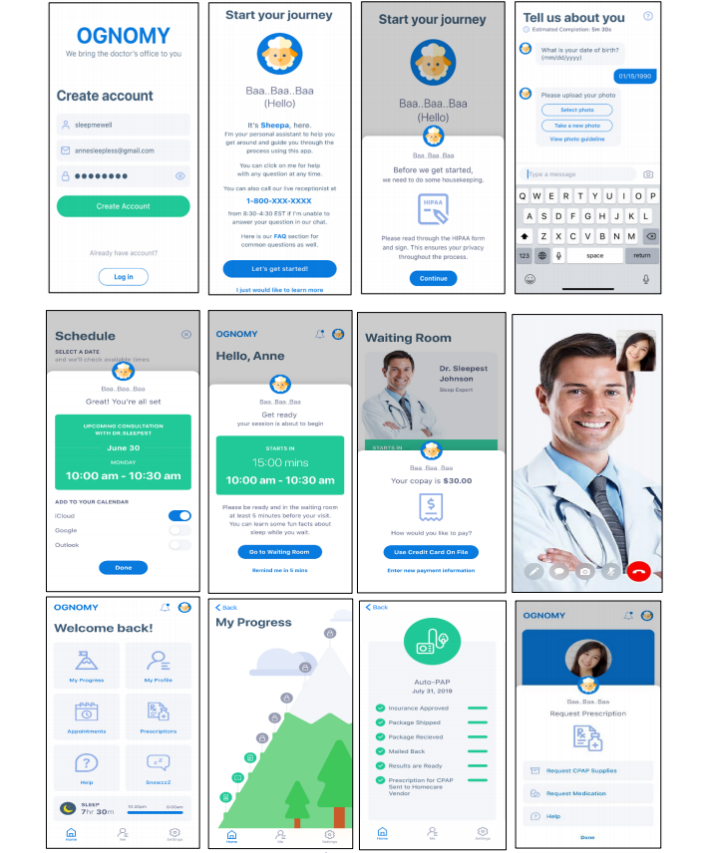
Mockup screens of the telemedicine platform.

To develop the product concept, we used iterative prototyping with varying levels of fidelity. In early iterations, the team developed paper prototypes and wireframes using the mock-up Marvel to seek early and continuous feedback from end users to improve the product concept. In the later iterations, the team finalized the mock-up screens of the product, as shown in Figure 3. Individuals intending to seek care for sleep apnea can download *Ognomy* from the Apple App Store or Google Play Store. Patients can create their accounts using email IDs and passwords. Subsequently, they can interact with our platform through an AI-based chatbot and furnish demographic, insurance, and medical information, as highlighted in screens 1-4. Thereafter, as presented in screens 5-8, patients can schedule an appointment with sleep specialists and will receive reminders 15 minutes before the appointment. Patients can then meet the sleep specialists virtually through video consultation, order a sleep test, review the results with their providers, and seek further treatment. At the other end, providers and sleep technicians used the web portal to offer their services. The dashboard provides sleep specialists and other providers with the opportunity to review patient data and monitor progress, as presented in screens 9-12. The platform is also integrated with EMRs and computerized physician order entry systems to enable seamless interaction among care providers and administrative staff.

#### Overall Technical Architecture

**Figure 4:**
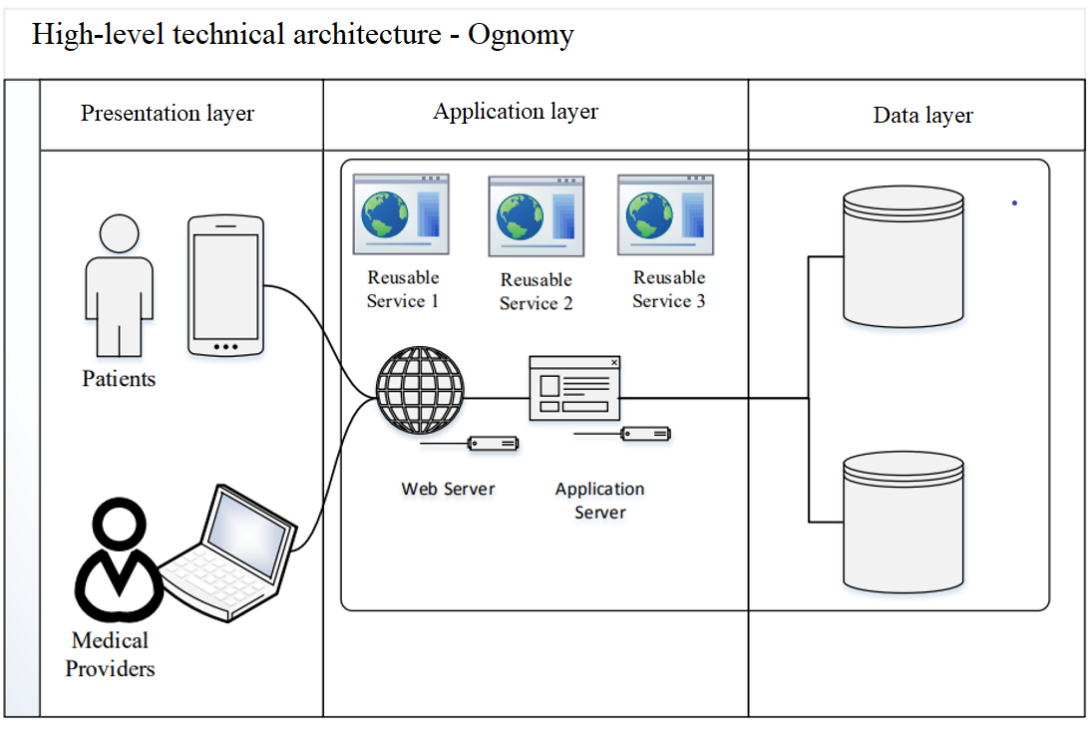
The technical architecture of the telemedicine platform.

We used a *layered modular architecture* that uses the software as a service app hosted in the cloud environment. This architecture leverages the capabilities of the best-of-breed apps to build a platform for a fee instead of developing them from scratch. For instance, to process out-of-pocket payments from patients, we can integrate with application programming interfaces offered by market leaders, such as Square, instead of developing our own payment processing apps. In addition to being robust [37], these services considerably reduce the software development time [38] and do not require a large upfront investment [39]. Given the high expectations in terms of interface quality and time to market, such an architecture was especially relevant for Ognomy. Figure 4 shows the high-level technical architecture used in this study. We used several third-party application programming interfaces, including AWS Cognito, and the latest technologies, such as React JS.

The clinical team, digital innovation consultants, and development team were dispersed across different geographies. The initial meeting to discuss the prospects of a digital sleep medicine platform occurred in June 2019. Ognomy was successfully deployed in the second quarter of 2020. The platform has been downloaded more than 4000 times and has received excellent reviews and ratings on Android and iPhone app stores. The platform has also been successfully deployed at two sleep centers, one in western New York and the other in Georgia. The sleep centers offer a full range of sleep apnea care and have diagnosed and treated more than 100,000 patients exclusive of those on the Ognomy platform.

### Evaluating Solutions and Generalizing Design Principles

#### Evaluation Strategy

**Figure 5:**
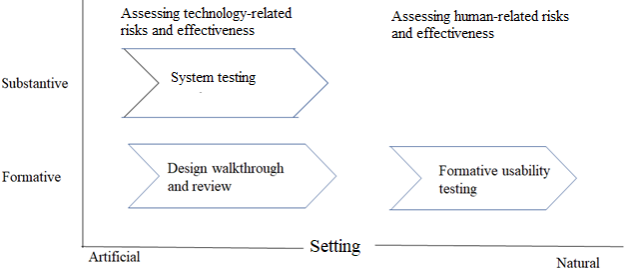
Evaluation approach.

Rigorous evaluation is a crucial activity in DSR that ensures the utility, quality, and impact of the resulting design artifacts. We followed the guidelines stipulated by the framework for evaluation in DSR [40] to evaluate the design artifact. We performed a formative evaluation by conducting an expert review of the technical design and system architecture to reduce technical risks. Furthermore, we assessed the platform summatively by performing end-to-end system testing to ensure the robustness of the artifact. We also assessed the platform’s effectiveness from a human perspective through formative field-based usability testing by the end users to identify and reduce user-related risks or concerns. We provide the details of our evaluation in Figure 5.

#### Design Review and Walkthrough

The evaluation of the design against the requirements was performed to ensure that the underlying system design was robust enough to support the product features. The review process is a preliminary activity that takes place before the development of the product begins. In their study, Tang and Lau [41] provided elaborate procedures for reviewing software architectures. In this study, we followed their approach to conducting a design review. The initial design was reviewed by 3 expert solution architects with more than 10 years of experience in data and system architectures. The details of the profiles are provided in Multimedia Appendix 2. The design was reviewed to ensure that there were no conflicts between (1) use context and design, (2) requirements and design, (3) design and development, and (4) design choice as compared with alternatives.

Furthermore, software design was reviewed against several technological aspects, such as service orientation, interface requirements, the platform’s core technology stack, environment details, security, backup, and performance best practices. The checklist used to perform the design review is provided in Multimedia Appendix 3. The findings of the design review were finalized by triangulating the reviews of the experts.

#### System Testing

To ensure a robust formative assessment of the product in an artificial setting, we conducted a system test on our platform. A test team of 3 expert software test professionals with an average software engineering experience of 4.35 years performed system testing. The team developed system test scenarios and test cases based on the requirements and design of the platform. Throughout the implementation phase, we followed an agile method of system testing where defects were identified and fixed on an iterative basis with regular product demonstrations on a weekly basis for 2 months. Given that the app was deployed in the production environment, all features were extensively tested. All the user interface screens, forms, actions, integrations, and end-to-end features, including positive and negative scenarios, were tested to ensure maximum test coverage. We also performed several compatibility tests with different handsets from iPhone and Android phones to ensure that the mobile app was consistent across the different devices.

Furthermore, we conducted several regression tests to ensure that the new code from the defect fixes did not introduce new defects inadvertently. Several defects were logged and fixed on an ongoing basis. Sample test cases and example defects are provided for reference in Multimedia Appendix 4. The system test phase concluded when critical patient-facing portions of the app, such as video consultation, appointment management, and reporting, were robust, with no critical or significant defects. However, addressing defects and issues in other parts of the apps was deferred for future releases.

#### Formative Field-Based Usability Testing

##### Overview

We conducted formative laboratory-based usability testing [42] on the fully functional mobile phone app prototype Ognomy to identify usability problems and obtain usability measures. Formative evaluations involve identifying and diagnosing problems, making and implementing recommendations, and then reevaluating the product, often iteratively, to detect and eliminate usability problems. In this study, we used the think-aloud approach to facilitate this process. After the participants provided consent to participate in the study, they were asked to complete a demographics form. Then, the participants were introduced to the think-aloud approach using a short video [43]. In this approach, the participants verbalized their thoughts and experiences as they moved through the app.

##### Subject Recruitment

We adopted a purposive sampling approach to select candidates for usability testing. We invited participants representing different stakeholder groups, including providers, patients, and hospital administrators. On the basis of their consent, the potential participants were invited to the testing center. Participants testing patient-facing apps were chosen depending on their risk profile, age, gender, education, and access to a mobile device. In this first version of the app, we intended to focus on the feedback from the critical segment of the target audience to ensure that our priorities, trade-offs, and design considerations were guided by the realities of individuals most likely to seek care for sleep apnea. Therefore, we decided to choose patients from the high-risk category. A total of 8 participants aged between 20 and 60 years who had access to a mobile device were included in the final sample.

Furthermore, to evaluate the apps’ physician-facing and administrative features, we adopted a convenient sampling approach. A total of 3 physicians and 3 hospital administrators with more than 10 years of experience in sleep and primary care were chosen to perform the evaluation. The details of the participants are provided in Multimedia Appendix 5.

##### Evaluation Design

After inviting the participants to the sleep laboratory, the subjects were introduced to the mobile or web app’s user interface design. The facilitator briefed the participants on the mobile or web app and informed them that they were evaluating the app and that the facilitator was not evaluating them. The participants signed an informed consent form acknowledging that participation was voluntary and that they could quit the study at any time. Although the sessions were audiotaped, the participants’ data privacy was ensured.

The facilitator instructed the participants to *think aloud* to obtain a verbal record of their interaction with the app. A mobile device and laptop with the web app and supporting software were used in a controlled environment. Each participant’s interaction with the app was monitored by a facilitator seated in the same office. The notetakers monitored the sessions. After all task scenarios were attempted, the participants completed the posttest satisfaction questionnaire regarding ease of use and satisfaction. These questionnaires were followed up with a debriefing at the end of the session.

##### Scenarios for the Patient

The participants went through typical scenarios on the app. The scenarios for would-be patient participants are listed in Textbox 1, and the scenarios for doctors and administrators are listed in Textbox 2.

**Scenarios for the patient.**

1. Create an account with your email address and answer all the questions asked by the artificial intelligence bot.
2. Schedule a consultation, follow through with the entire process, and then reschedule an appointment for another day. Schedule the appointment from the account created in task 1 and receive a reminder for the consultation. Then, reschedule the appointment.
3. Consult with a doctor on a video call: attend the consultation using a video call and validate the various options, including notes, messages, with the camera on or off, decrease or increase volume, and mute.
4. Postconsultation comments and order a home sleep test: review the doctor’s comments and select from the given choices to order a home sleep test and perform the test.
5. Refer a patient and provide the patient’s information. Provide a friend’s information as the referring patient.

**Scenarios for doctors and administrators.**

- Check your appointments: see the upcoming appointments this week or month and review recent sessions.
- Review the to-do list: check or add reminders and tasks, make notes, and see new messages from patients, secretary, or office.
- Consult with a doctor on a video call: attend the consultation using a video call and check the notes and messages, explore camera options, decrease or increase volume, mute, and turn the camera off.
- Study the current treatments of the patients: bring up the details of the treatment provided to the patients, including the notes which they made about it, and update the information.

##### Posttest Questions

To further understand the perceptions of the users, we conducted posttest interviews following the best practices suggested by usability testing guides and exemplars [44,45]. The questions are listed in Textbox 3.

**Posttest questions.**

1. How would you describe the app to someone?
2. What was your favorite aspect of the app?
3. What was the most confusing part of the app?
4. Would you continue using this product? If so, why?
5. Would you recommend this product to a friend or colleague?

## Results

Participants in the usability studies highlighted several interesting issues and classified them as critical or noncritical. The compiled list of issues, along with the steps taken for their resolution, are presented in Tables 2 and 3.

Overall, we received positive feedback from all types of participants on visual design aspects, such as color choices, esthetic appeal, the contrast ratio between font and the background, and the information architecture of the product. However, patients and administrative users highlighted some issues with the onboarding workflow and patient profiles. The crux of the problem was the large volume of information, including personal information, medical information, insurance information, and payment information, that patients had to furnish before setting up an appointment with a physician. They also suggested several enhancements to the onboarding workflow, such as adding a progress bar, providing a guided tour, and moving some of the information uploads to postappointment screens. Physician users, on the other hand, were mainly concerned about calendar design and notification issues.

We addressed all critical and noncritical feedback in the patient-facing parts of the app before launching the product. Several prior studies have emphasized the importance of patient-centered design as a prerequisite for the adoption and eventual success of mobile health apps [46]. Therefore, we deliberately prioritized patient-specific issues to develop a patient-first platform. Except for showstopper defects, the issues and concerns of other users, including administrators and providers, were deprioritized for the current version and deferred for later releases.

**Table 2:** Critical issues from usability tests.

ID	Stakeholder	Critical issues	Steps taken for their resolution
1	Patient or admin	Simpler customer onboarding process	Two important changes were made as part of the onboarding process. First, registration process was simplified to include only basic fields, such as name, email, and password. Second, chatbot-based interaction during data collection was enhanced to include optical character recognition capability to simplify data entry for patients.
2	Patient	Unavailability of user profile: there is no option that allows the user to access their profile page and edit the details in just a single click. This is especially important when the users provide incorrect information to the bot by mistake.	A patient dashboard and account home page were developed to resolve this concern.
3	Patient	Status indicator: several studies have highlighted that users are more motivated to complete the tasks as they get closer to the end. A progress bar that indicates the status would better engage the users.	The issue was resolved through front-end code changes.
4	Patient	Option to exit from the app: users should have an option to exit from any screen in the app if they so choose. For instance, if a user opts to book an appointment later, the user should have the ability to do so.	User interface–related code changes were made to provide users an exit option from any screen. At the backend, information provided by the users during each session would be stored.

**Table 3:** Noncritical issues from usability tests.

ID	Stakeholder	Noncritical issues	Steps taken for their resolution
1	Admin	Guided tour: the app does not have an initial tour guide. Having a tour guide would ease the user into onboarding, facilitate smarter user training, and reduce support queries with interactive walkthroughs.	A context-specific support is integrated with the artificial intelligence bot to facilitate user training and reduce support cost.
2	Physician or admin	UI^a^ calendar’s click: the app does not allow users to navigate dates with minimal clicks. One must click multiple times to reach dates after a few months or years.	Two resolutions were provided. First, the navigation across dates was made easy by providing users month and year pickers in addition to the day. Second, a provision to type in the date was provided to the users.
3	Admin	Rewording referral functionality: currently, the functionality of referring a friend is labeled as “Patient Referral,” which is confusing. It should be labeled “Refer a Friend.”	We intend to fix the label with code changes in the UI programs; however, the issue has been deferred to a future version.

^a^UI: user interface.

## Discussion

### Theoretical Implications

This design science study makes several notable contributions to both theory and practice. We make several theoretical contributions of this study. Although ISs research in the health care field has been ripe with development [47], there have not been many interventions developed for patients seeking sleep apnea care [48]. Furthermore, extant DSR has mostly emphasized the design, development, and evaluation of IT solution artifacts [49]. Exploring and validating the problem space have been insufficiently pursued. In this study, we took advantage of the design knowledge model [50] to bifurcate the design space into problem and solution spaces. Problem space validation establishes the relevance of the research product, whereas solution space addresses technical feasibility and product usability.

This study also informs the ISs research community on how telemedicine interventions can be implemented using the activity theory framework for building and evaluating artifacts. Activity theory in DSR has been used to design data models [51] and mobile apps in an educational context [52]. In this study, we enhanced activity theory by mapping the business activity system to a problem space and the technical activity system to a solution space. Furthermore, we argue that any IT artifact embodies the social and technical aspects of system development. The success of an artifact depends on the interactions between business and technical activity systems.

### Practical Implication

From a practice perspective, we designed and developed a telemedicine platform and demonstrated the potential for such an intervention. We also showed how novel methods for managing products and developing digital architecture can quickly and effectively scale digital interventions. This study generated transferable insights into the most effective practices in conceptualizing and developing digital health products. A total of four core themes of generalizable guidelines emerged:

- learning from diverse end user perspectives is critical
- the use of frugal engineering methods to foster cost savings and reduce the time to market can be highly effective
- the commoditization of software through layered modular architecture and cloud-based infrastructure is the new norm
- access to software development talent

We have provided an elaborate discussion on these themes in the following sections.

### Learning From Diverse End User Perspectives Is Critical

As highlighted in several prior studies, software development organizations may be tempted to quickly cruise through the conceptualization and design phases without validating the problem space from an end user perspective [53]. However, such practices usually lead to the development of products that are not grounded in their customers’ needs. Researchers frequently use co-design workshops and structured interviews with different stakeholders to overcome this challenge [54].

In this study, we note that the diversity of opinions early on is especially important, as it provides a good platform for all ideas to emerge. It facilitates the holistic development of an app that is relevant, usable, and valuable to all stakeholders, including patients and clinicians, thus overcoming potential barriers to successful adoption and continued use. This implication is particularly relevant for complex business domains, such as health care, where several subject matter experts, such as medical providers (doctors and nurses), administrators (billing, coders, and operation managers), and sleep technicians, interact with end users (patients).

### The Use of Frugal Engineering Methods to Foster Cost Savings and Reduce the Time to Market Can Be Highly Effective

Extant literature has emphasized that weeding out feature ideas without potential quickly and economically can be a critical success factor in the development of digital platforms [55,56]. To accomplish this goal, development organizations must leverage a combination of novel frugal engineering methods to save time and money. For instance, in our study, we used iterative prototyping with varying fidelity levels to understand the potential problems before building the final product.

### The Commoditization of Software Through Layered Modular Architecture and Cloud-Based Infrastructure Is the New Norm

The team used layered modular architecture using the software as a service app hosted on the cloud environment. Such architecture helps borrow capabilities from other apps for a fee instead of developing them from scratch. In addition to being robust, these services reduce the development time considerably and do not require a large upfront investment. Furthermore, the app was hosted on the AWS cloud environment, which complied with all technology-related HIPAA requirements. The team also used tools such as EC2 (Elastic Compute Cloud), S3 (Simple Storage Service), and ELB (Elastic Load Balancer) to enhance the ease of deployment and migration and to meet on-demand scaling.

### Access to Software Development Talent

Prior research has highlighted the shortage of software development talent [57]. This challenge is pronounced in nontechnology hubs. Although many universities offer courses in software engineering and several boot camps and training centers have arisen to fill the talent gap, the availability of solid software development talent, especially in the latest technologies such as React JS and dev-ops automation that can deliver apps quickly in short time frames, remains a significant bottleneck.

### Limitations

This study had several limitations. We conducted formative usability tests with only a limited number of end users, including 8 patients, 3 providers, and 3 sleep clinic administrators. As a result, the issues identified in the usability tests may not be generalizable to other settings. Furthermore, all the participants of the usability tests were from the northeastern United States, and hence, the sample of participants may not be nationally representative. However, our focus was not on the generalizability of our findings but on gathering practical insights about the challenges faced by the end users of the app.

Although the participants testing the patient-facing apps exhibited diversity in age, gender, and educational accomplishments, they all had access to smartphones and were reasonably comfortable using them. Our findings might have been significantly different if we had focused our attention on individuals who lacked technology proficiency or who had cognitive or physical challenges. However, given the ubiquity of mobile devices and the large number of smartphone users, we deliberately focused on this market segment.

Another limitation is that not all the issues identified during the design review, system test, and formative usability tests were addressed in the current version of the app. We refined the design artifact based on the priority of the concerns raised, as we were constrained by time and budget. All the critical issues and errors associated with the patient-facing features, such as appointment management and video consultation, were addressed. However, issues associated with EMRs and computerized physician order entry integration; physician reports; and administrative features, such as insurance card verification, were deferred for future releases.

### Conclusions

Against the backdrop of a surge in demand for sleep apnea care, the telemedicine platform offers a scalable and economical alternative. With an aging population and the escalating cost of care, telemedicine alternatives have become increasingly imperative. This is especially true for patients seeking care for sleep apnea because of its increasing prevalence. This paper discusses the design, development, and evaluation of a telemedicine intervention that aids providers and care seekers in virtual consultation, conducts tests, and prescribes treatment virtually. Such a telemedicine platform breaks access barriers while ensuring high-quality care. During the evaluation phase, we noted that this platform is economically viable, technically feasible, and highly usable by all stakeholder groups. Future research can extend this line of inquiry by developing artifact instances for other sleep-related issues, such as insomnia. An interesting contribution would be to demonstrate how telemedicine interventions are accepted across different cultures and geographies, especially where there is a lack of awareness about sleep issues. Future work could also address related issues of e-consent and explore the legal and ethical guidelines for using such platforms.

## Appendix

Multimedia Appendix 1 Questionnaire for requirements gathering interviews.

Multimedia Appendix 2 Expert profiles for design and architectural review.

Multimedia Appendix 3 Checklist for design and architectural review.

Multimedia Appendix 4 Sample defects found during system testing.

Multimedia Appendix 5 Participant profiles.

## Abbreviations

AI: artificial intelligence
CPAP: continuous positive airway pressure
DSR: design science research
EC2: Elastic Compute Cloud
ELB: Elastic Load Balancer
EMR: electronic medical record
HIPAA: Health Information Portability and Accountability Act
IS: information system
IT: information technology
OSA: obstructive sleep apnea
S3: Simple Storage Service
