# Supplementary material for: Design, Development, and Evaluation of a Telemedicine Platform for Patients With Sleep Apnea (Ognomy): Design Science Research Approach
Source: JMIR Form Res. 2021 Jul 19;5(7):e26059. doi: 10.2196/26059 (PMC8329758; doi:10.2196/26059)
Supplement: Multimedia Appendix 1 [file formative_v5i7e26059_app1.docx]

Appendix- 1

**Questionnaire for requirements gathering interviews.**

**High Level questions**

- What do you intend to accomplish with the platform?
- Who are the key stakeholders and users? Do their goals differ? If so, how?
- How do the project/system’s goals map to the department goals or needs?
- What information do you need from this project/system that you don’t have now?
- Are there other projects/systems with which this project/system will interface?
- Is there any existing project/system documentation? If so, where? Who else should I talk to?

**Current Needs**

- What department/business requirements will this project/system address?
- Is any of this data currently captured in any other project/system? Is the data and/or functionality shared by others?

**Current Problems**

- Do you have to do things manually that you would like to automate?
- Do you have performance problems that need to change? Do you have functional limitations that you’d like to change?

**Criteria for Success**

- What needs to happen to make this project/system successful?
