# Supplementary material for: Design, Development, and Evaluation of a Telemedicine Platform for Patients With Sleep Apnea (Ognomy): Design Science Research Approach
Source: JMIR Form Res. 2021 Jul 19;5(7):e26059. doi: 10.2196/26059 (PMC8329758; doi:10.2196/26059)
Supplement: Multimedia Appendix 2 [file formative_v5i7e26059_app2.docx]

## Appendix 2

## Expert profiles for design and architectural reviews

1

Informant #1 is a Senior data architect with 13 years of health IT products. The Informant is currently employed with as an independent consultant with several health care organizations. Informant #1 brings expertise in enterprise and data architecture and holds several enterprise and data related certifications including Certified Enterprise Architect (CEA), AWS solution architect – associate among others.

2

Informant #2 is a Lead Application Developer (Full-Stack) at a Teaching hospital of a university in the Northeast of the United States. Informant #3 has 11 years in Health IT at the current employer and is currently in charge of application integration. The informant holds several health IT certifications including the Certified Scrum Master (CSM), and the Health Level 7 (HL7) Control Specialists certifications.

3

Informant #3 is a lead architect with over 15 years of data architecture experience. Certified Enterprise Architect (CEA) focusing on the Zachman Framework, The Federal Enterprise Architecture Framework (FEAF) and the Department of Defense Architecture framework (DODAF).
