# Supplementary material for: Design, Development, and Evaluation of a Telemedicine Platform for Patients With Sleep Apnea (Ognomy): Design Science Research Approach
Source: JMIR Form Res. 2021 Jul 19;5(7):e26059. doi: 10.2196/26059 (PMC8329758; doi:10.2196/26059)
Supplement: Multimedia Appendix 3 [file formative_v5i7e26059_app3.doc]

Appendix 3

**Checklist for design and architectural review**

|  | | | | |
| --- | --- | --- | --- | --- |
| **Checklist Description:**  This checklist captures common elements that should be present in any design. It is presented during the Design Review process to stimulate thought, guide brainstorming, and to ensure the design being outlined contains all proper design considerations. As the project architecture, system, and application design is being reviewed, assess the design considerations that apply to your subject matter expertise and business/technical needs. | | | | |
| **Project Name:** | | **Review Date:** | | |
| **Assessment and Recommendations:**  Approved without revision  Approved with revisions (see Notes)  Not approved | | **Notes:** | | |
| **Reviewer:** | | | **Signature:** | |
| **Artifacts Reviewed:**  Technical Design Specification  Implementation Plan | | | Conceptual Architecture Review Checklist  Requirements Traceability Matrix | |
| **General Design** | | | | **Comments** |
|  | Does the design support both product and project goals? | | |  |
|  | Is the design feasible from a technology, cost, and schedule standpoint? | | |  |
|  | Have known design risks been identified, analyzed, and planned for or mitigated? | | |  |
|  | Are the methodologies, notations, etc. used to create and capture the design appropriate? | | |  |
|  | If possible, were proven past designs reused? | | |  |
|  | Does the design support proceeding to the next development step? | | |  |
|  | Have proper fallback consideration been made? | | |  |
| **Design Considerations** | | | | **Comments** |
|  | Does the design have conceptual integrity (i.e., does the whole design tie together)? | | |  |
|  | Can the design be implemented within technology and environmental constraints? | | |  |
|  | Does the design use standard techniques and avoid exotic, hard-to-understand elements? | | |  |
|  | Is the design unjustifiably complex? | | |  |
|  | Is the design lean (i.e., are all of its parts strictly necessary)? | | |  |
|  | Does the design create reusable components if appropriate? | | |  |
|  | Will the design be easy to port to another environment if appropriate? | | |  |
|  | Does the design allow for scalability? | | |  |
|  | Are all time-critical functions identified, and timing criteria specified for them? | | |  |
|  | Are the hardware environment completely defined, including engineering change levels and constraints? | | |  |
|  | Are the pre-requisite and co-requisite software and firmware clearly identified, including release levels and constraints? | | |  |
| **Requirements Traceability** | | | | **Comments** |
|  | Does the design address all issues from the requirements? | | |  |
|  | Does the design add features or functionality, which was not specified by the requirements (i.e., are all parts of the design traceable back to requirements)? | | |  |
|  | If appropriate, has requirements coverage been documented with a completed requirements traceability matrix? | | |  |
|  | Are all of the assumptions, constraints, design decisions, and dependencies documented? | | |  |
|  | Have all reasonable alternative designs been considered, including not automating some processes in software? | | |  |
|  | Have all goals, tradeoffs, and decisions been described? | | |  |
|  | Has the Risk Plan been modified with any new risks posed by the design? | | |  |
|  | Have all interfacing systems been identified? | | |  |
|  | Are the error recovery and backup requirements completely defined? | | |  |
|  | Have the infrastructure e.g. backup, recovery, checkpoints been addressed? | | |  |
| **Consistency** | | | | **Comments** |
|  | Does the design adequately address issues that were identified and deferred at previous upstream levels? | | |  |
|  | Is the design consistent with related artifacts (i.e., other modules, designs, etc.)? | | |  |
|  | Is the design consistent with the development and operating environments? | | |  |
| **Performance Reliability** | | | | **Comments** |
|  | Are all performance attributes, assumptions, and constraints clearly defined? | | |  |
|  | If appropriate, are there justifications for design performance (i.e., prototyping critical areas or reusing an existing design proven in the same context)? | | |  |
| **Capacity Planning** | | | | **Comments** |
|  | Does the design improve productivity? | | |  |
|  | Is scalability development into the plan and is maintainable? | | |  |
|  | Is Total Cost of Ownership (TCO) controlled or reduced? | | |  |
| **Maintainability** | | | | **Comments** |
|  | Does the design allow for ease of maintenance? | | |  |
|  | If reusable parts of other designs are being used, has their effect on design and integration been stated? | | |  |
|  | Does the design resist erosion in the correctness of its content over time? | | |  |
| **Compliance** | | | | **Comments** |
|  | Does the design follow all standards necessary for the system? (i.e., date standards) | | |  |
|  | Have legal/regulatory requirements been assessed and accounted for? | | |  |
| **Modeling and Design Views** | | | | **Comments** |
|  | When appropriate, are there multiple, consistent, models and/or views that represent the design (i.e., static vs. dynamic)? | | |  |
|  | Where there are multiple models of the software (i.e., static and dynamic) are those models consistent with each other? | | |  |
