# Supplementary material for: Design, Development, and Evaluation of a Telemedicine Platform for Patients With Sleep Apnea (Ognomy): Design Science Research Approach
Source: JMIR Form Res. 2021 Jul 19;5(7):e26059. doi: 10.2196/26059 (PMC8329758; doi:10.2196/26059)
Supplement: Multimedia Appendix 4 [file formative_v5i7e26059_app4.docx]

Appendix 4

**Sample defects found during system testing.**

- **Navigation to Home screen:** Currently, there is no option that allows the user to access the home page with a single click.
- **Booking an appointment based on doctors/patient's availability:** The user should be able to choose the time slot based on their availability and the doctor's availability.
- **User History**: Users must be able to preserve a record of their previous appointments, which will include details like Bill summary, date and time of the appointment, chat, prescription.
- **Email**: There should be a functionality that sends the information related to the consultation on emails which will provide users easy accessibility to the documents and information needed.
- **In-app Notification**: The content of in-app notification will help highlight important information to the user, like reminding the user of completing the boarding process or notifying them of an upcoming appointment.
