# Supplementary material for: Design, Development, and Evaluation of a Telemedicine Platform for Patients With Sleep Apnea (Ognomy): Design Science Research Approach
Source: JMIR Form Res. 2021 Jul 19;5(7):e26059. doi: 10.2196/26059 (PMC8329758; doi:10.2196/26059)
Supplement: Multimedia Appendix 5 [file formative_v5i7e26059_app5.docx]

Appendix 5

**Participants profiles for usability testing***

| Id | Participant Description – End users (Patients) |
| --- | --- |
| 1 | 33-year-old, Male, Bus Driver, BMI – 27.3, Caucasian |
| 2 | 28-year-old, Female, Graduate Student, BMI – 24.2, Asian |
| 3 | 53-year-old, Female, Financial Officer, BMI – 21.8, Caucasian |
| 4 | 22-year-old, Male, Undergraduate Senior, BMI – 30.2, Asian |
| 5 | 35-year-old, Female, Homemaker, BMI- 26.7, Asian |
| 6 | 44-year-old, Female, Teacher, BMI-24.4, African American |
| 7 | 54-year-old, Male, IT executive, BMI – 27.8, Caucasian |
| 8 | 23-year-old, Female, Graduate student, BMI – 22.8, African American |

| Id | Participant Description - Providers |
| --- | --- |
| 1 | Nurse practitioner with 10 years of experience in sleep clinics |
| 2 | 16 Years of Primary care experience with substantial research work in patient safety issues |
| 3 | Nurse practitioner with 8 years of clinical experience. Currently pursuing a master’s in health informatics. |

| Id | Participant Description – Hospital Administrator |
| --- | --- |
| 1 | Practice director with 13 years of experience in a large clinic |
| 2 | Practice director with 10 years of experience in a large clinic |
| 3 | Billing and coding consultant with 7 years of experience |

*BMI was computed based on the self-reported height and weight of the participants.
